# Hernia Testis from Strumous Deposit

**Published:** 1860-04

**Authors:** 


*Hernia Testis from Strumous Deposit. Partial removal: Cure.*—The strumous diathesis is one of the most unfavorable complications under which inflammatory affections of the testicle can occur. A blow on the testicle, which, in a healthy individual, excites only transient or trifling orchitis, will, in a strumous constitution, light up chronic disease, accompanied by enduring enlargement and strumous deposit; frequently at a latter stage this strumous matter breaks down, abscess forms, and hernia testis in its most troublesome aspect is the result.

Of this form of the disease the following case afforded a good type:—J. A—, aged forty-five, was admitted into St. Mary's Hospital, under the care of Mr. Coulson, having the scrotum greatly enlarged, and swollen to the size of a shaddock, tense and elastic to the touch. From an aperture on the right side, the corresponding testis protruded, forming a shining, irregular mass, of the size of a walnut, adherent to the surface of the scrotum. The disease dated from a sharp blow received six month's previously, subsequent to which occurrence there were pain and swelling to a considerable degree; this subsided almost wholly under appropriate medical treatment; the scrotum had never resumed its former size, but remained somewhat large and indurate. Four weeks before admission, the swelling gradually increased, and about a week before, the skin gave way, and the testicle came into view. Mr. Coulson made two punctures in the scrotum in order to evacuate its fluid contents, and employed compression and suspension. Under this treatment the swelling greatly diminished; but the protrusion of the testis persisted. He determined, therefore, to perform ablation of the fungoid portion of the testis, and to return the remaining part. He proceeded to dissect the testis from the scrotum, enlarged by upward and downward incisions the aperture of protrusion, removed that part of the testis which was infiltrated with strumous deposit, and returned the healthy portion into the scrotum; bringing, then, the clean edges of the wound together by many points of suture. The patient did perfectly well for some days. A sharp attack of erysipelas then ensued; the scrotum, groin, and perinæum were inflamed; the wound gaped, and the testis protruded: the scrotum became hard and thickened, and the skin livid. Under appropriate treatment, however, the erysipelatous inflammation subsided. Mr. Coulson then employed tonics, and a lotion of five grains of nitrate of silver to one ounce of lime water. Under this treatment the scrotum contracted around the testis, so that when cicatrization was completed, and the parts had healed, the testis was completely covered, and the man was discharged cured.

On the 1st inst., we were present at the London Hospital, when Mr. Curling dissected the skin around a benign fungus of the testicle in a colored man, and brought it over the protruding mass, which comprised nearly the whole of the testicle. This was the result of chronic orchitis, but the bulk of the tumor had become diminished through absorption from the internal use of the iodide of potassium.—*London Lancet.*

